# Exploitation of reverse vaccinology and immunoinformatics as promising platform for genome-wide screening of new effective vaccine candidates against *Plasmodium falciparum*

**DOI:** 10.1186/s12859-018-2482-x

**Published:** 2019-02-04

**Authors:** Manisha Pritam, Garima Singh, Suchit Swaroop, Akhilesh Kumar Singh, Satarudra Prakash Singh

**Affiliations:** 1 Amity Institute of Biotechnology, Amity University Uttar Pradesh, Lucknow Campus, Lucknow, 226028 India; 2 Department of Zoology, University of Lucknow, Lucknow, 226007 India

**Keywords:** Malaria, Antigen, Epitopes, Population coverage, Vaccine, *Plasmodium falciparum*

## Abstract

**Background:**

In the current scenario, designing of world-wide effective malaria vaccine against *Plasmodium falciparum* remain challenging despite the significant progress has been made in last few decades. Conventional vaccinology (isolate, inactivate and inject) approaches are time consuming, laborious and expensive; therefore, the use of computational vaccinology tools are imperative, which can facilitate the design of new and promising vaccine candidates.

**Results:**

In current investigation, initially 5548 proteins of *P. falciparum* genome were carefully chosen for the incidence of signal peptide/ anchor using SignalP4.0 tool that resulted into 640 surface linked proteins (SLP). Out of these SLP, only 17 were predicted to contain GPI-anchors using PredGPI tool in which further 5 proteins were considered as malarial antigenic adhesins by MAAP and VaxiJen programs, respectively. In the subsequent step, T cell epitopes of 5 genome derived predicted antigenic adhesins (GDPAA) and 5 randomly selected known malarial adhesins (RSKMA) were analysed employing MHC class I and II tools of IEDB analysis resource. Finally, VaxiJen scored T cell epitopes from each antigen were considered for prediction of population coverage (PPC) analysis in the world-wide population including malaria endemic regions. The validation of the present in silico strategy was carried out by comparing the PPC of combined (MHC class I and II) predicted epitope ensemble among GDPAA (99.97%), RSKMA (99.90%) and experimentally known epitopes (EKE) of *P. falciparum* (97.72%) pertaining to world-wide human population.

**Conclusions:**

The present study systematically screened 5 potential protective antigens from *P. falciparum* genome using bioinformatics tools. Interestingly, these GDPAA, RSKMA and EKE of *P. falciparum* epitope ensembles forecasted to contain highly promiscuous T cell epitopes, which are potentially effective for most of the world-wide human population with malaria endemic regions. Therefore, these epitope ensembles could be considered in near future for novel and significantly effective vaccine candidate against malaria.

**Electronic supplementary material:**

The online version of this article (10.1186/s12859-018-2482-x) contains supplementary material, which is available to authorized users.

## Background

The human malaria is triggered as a result of 5 species of *Plasmodium* protozoan parasite (*P. falciparum*, *P. vivax*, *P. ovale*, *P. malariae* and *P. knowlesi)*. However, *P. falciparum* is one of the most deadly species that responsible towards > 90% of overall malaria mediated deaths. On the basis of 2016 data, WHO evaluated that almost 445,000 deaths were due to malaria with a total of 216 million cases from 91 countries. The African region (15 countries), continues to account for about 91% of malaria deaths worldwide including 80% by sub-Saharan Africa as well as virtually all of the remaining incidence reported from South-East Asia and the Indian sub-continent with South America. Thus, if we want to get the global response of malaria vaccine, these heavily affected countries in African region must be our primary focus [1].

Human infection with the malaria parasite is developed following the inoculation of the sporozoite stage of the protozoan parasite by female anopheles mosquitoes. Infants and young children in malaria-endemic countries of African region naturally come across numerous clinical episodes of malaria before they build up partial immunity that defends towards severe disease as well as malaria mediated death. The mechanisms related to naturally acquired immunity are not completely explored; nevertheless, there are two foremost theories. First is the gradual attainment of strain-specific immunity, while second is the recurring antigenic exposure, possibly in combination with an age-linked immune maturation [2]. Although, important roles of both humoral as well as cell-mediated immune responses were demonstrated in animal models and humans subsequently natural malaria infection and exposure to experimental malaria vaccines. However, no clear correlation for protection have been documented for existing vaccine candidates except antibodies against circumsporozoite protein (CSP), which depict some correlation for protection towards the pre-erythrocytic stages of the parasite [3, 4].

Although, there are more than 30 vaccine candidates of malaria that were involved in different clinical phase trail, but they did not make a globally effective vaccine [5, 6]. The RTS,S is latest leading vaccine candidate with partial protection efficacy (39.0% in clinical malaria and 20.5% in severe malaria case) and restricted in limited regions of African countries [7]. The RTS,S is a recombinant vaccine of CSP and Hepatitis B surface antigen with liposomal adjuvant [5, 8], which induces the anti-CSP antibodies and CD4^+^ T cells during phase-III clinical trials [3, 4]. However, CD8^+^ T cell response remain missing from the RTS,S vaccine. Moreover, the attainment of naturally acquired partial immunity against malaria infection and some correlation to protection against experimental malaria vaccines, provide a positive perception towards the design of globally efficient malaria vaccines. Nonetheless, the large size of the *Plasmodium* genome (23.3 Mb) with more than 5000 genes pose significant challenges in experimental identification of immunodominant epitopes for activating both CD4^+^ and CD8^+^ T cells.

In past two decades, as a result of development of reverse vaccinology and immunoinformatics, identification of antigen-specific CD4^+^ and CD8^+^ T cell epitopes turn out to be more straightforward approach along with less laborious and low cost [9–14]. Concisely, this approach is based on the screening of antigenic features from the genome sequence of pathogen and further prediction of peptide ligands, which establish stable complexes (high affinity) with major histocompatibility complex (MHC) molecules. These MHC-peptide complexes can be used to monitor antigen-specific CD4^+^ and CD8^+^ T cell responses [15, 16]. Hence, the finding of targets for protective immunity has been the sole utmost significant objective of the overall immunologist involved towards the design and improvement of anti-malarial vaccines [17]. Critical review of literature depicted that there is little research work has been carried related to population coverage analysis of predicted and experimentally known epitopes (EKE) of *P. falciparum* [18].

In the present work, we hypothesized that application of reverse vaccinology along with immunological bioinformatics tools might uncover *P. falciparum*-resulting epitopes specific for CD4^+^ and CD8^+^ T cells involved in world-wide protection towards malaria. This study focus on genome-wide in silico screening of putative antigens of *P. falciparum* 3D7 genome and further prediction of human leukocyte antigen (HLA) class I and II binding epitopes covered by major world-wide population including malaria endemic regions through immune epitope database (IEDB) based prediction of population coverage (PPC). Ultimately, the predicted epitope ensemble of genome derived predicted antigenic adhesins (GDPAA) showed considerably higher world-wide population coverage (99.97%) compared to randomly selected known malarial adhesins (RSKMA) (99.90%) and EKE of *P. falciparum* (97.72%). These predicted epitope ensembles could be considered as promising candidate for effective nano vaccine design against malaria.

## Methods

### Data resources

The whole protein sequences (5548) of *P. falciparum* 3D7 genome were retrieved from PlasmoDB database (http://plasmodb.org) into the FASTA format, accessed on October 3, 2017 together with 5 RSKMA. Similarly, 151 non redundant EKE of *P. falciparum* 3D7 including 121 MHC class I and 30 MHC class II were retrieved from IEDB database (www.iedb.org) accessed on February 20, 2018 using MHC biding assay as search criteria (Additional file 1).

### Methodology of the work

**Fig. 1:**
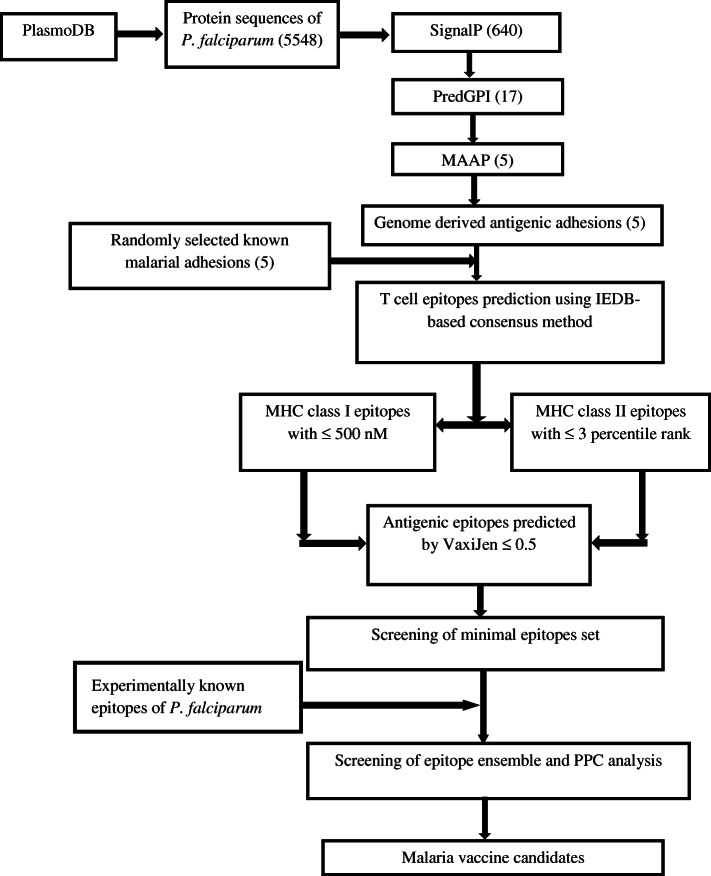
Methodology flow chart used in the present study for genome-wide screening of effective malaria vaccine candidates

This investigation facilitates the screening of vaccine candidates from entire protein sequences (5548) of *P. falciparum* genome involved three major steps: i) prediction of probable antigens ii) prediction of T cell epitopes iii) screening of minimal epitope ensemble for maximum human population coverage. The methodology flow chart is given in Fig. 1.

### Prediction of antigens and physico-chemical characterization

The first step involved hypothetical confirmation for occurrence of surface protein and antigenic features assessed from protein primary sequences employing well-known tools: SignalP4.0, PredGPI, MAAP, VaxiJen and TMHMM in sequential manner to identify probable antigens [19, 20]. The proteins of *P. falciparum* were reflected as high-grade antigen candidates if they have forecasted features of a surface protein, i.e., a signal peptide, a glycophosphatidylinositol (GPI) anchor and transmembrane domain(s). Currently, SignalP4.0 is the most advanced and widely used tool for prediction of signal peptides from amino acid sequences of protein. The protein with signal peptide is targeted to the secretory route, nevertheless it is not necessarily to be secreted [21]. Numerous eukaryotic proteins are accompanying with the extracellular leaflet of the plasma, which carries a GPI-anchor associated with the C-terminal residue after a proteolytic cleavage occurring at omega-site [22]. The position of the GPI omega site within the secretary route proteins was predicted using PredGPI tool [23]. Surface proteins having functions in immune evasion and/ or cytoadhesion, are of importance also as promising vaccine candidates. Adhesion of *Plasmodium* parasites is mediated by proteins called adhesins. The parasite can be inhibited by immunizing the host with adhesins. Therefore, these GPI-anchored surface proteins were screened as malarial adhesins and adhesin-like proteins by MAAP tool [24–26]. The VaxiJen2.0 program was used for the evaluation of antigenicity of the malarial adhesins protein sequence [27, 28]. Furthermore, to assess the potential impact of membrane proximity, transmembrane regions of potential antigens (pathogenesis-related secretome of *P. falciparum*) were predicted using TMHMM2.0 tool [29–31]. The physico-chemical characterization [(grand average of hydropathicity (Gravy), theoretical isoelectric point (pI)] and water solubility analysis of all the 5 GDPAA and 5 RSKMA were studied by using tools ExPASy-ProtParam and INNOVAGEN, respectively [32–34]. Moreover, the clustering tree analysis of all the 5 GDPAA was carried out by MAFFT7.0 web server. In this context, the detail web addresses and threshold criteria of in silico tools exploited in the current investigation are provided in Table 1.

**Table 1:** Details of the computational tools used for the prediction of antigens, epitopes and their population coverage analysis

S.No.	Database/tool name	Description	URL	Threshold criteria
1	SignalP4.0	Presence and location of signal peptide cleavage sites	http://www.cbs.dtu.dk/services/SignalP/	Default
2	PredGPI	Prediction system for GPI-anchored proteins	http://gpcr.biocomp.unibo.it/predgpi/pred.htm	Default
3	MAAP	Prediction of Malarial adhesins and adhesins-like proteins.	http://maap.igib.res.in/	≥ 0
4	TMHMM2.0	Transmembrane helices in the integral membrane proteins	http://www.cbs.dtu.dk/services/TMHMM/	≤ 1
5	VaxiJen2.0	Prediction of protective antigens	http://www.ddg-pharmfac.net/vaxijen/VaxiJen/VaxiJen.html	≥ 0.5
6	ExPASy-ProtParam	Calculation of physicochemical parameters	https://web.expasy.org/protparam/	Default
7	MAFFT7.0	Multiple sequence alignment	http://mafft.cbrc.jp/alignment/server/large.html.	Default
8	INNOVAGEN	Prediction of water solubility of proteins	https://pepcalc.com/	Default
9	IEDB-MHC class I-Consensus	Prediction of MHC class I binding epitopes	http://tools.iedb.org/mhci/	IC_50_ ≤ 500 nM
10	IEDB-MHC class II-Consensus	Prediction of MHC class II binding epitopes	http://tools.iedb.org/mhcii/	≤ 3 percentile rank
11	IEDB-Population Coverage	Population coverage analysis of selected epitopes	http://tools.iedb.org/population/	Default
12	CTLPred	Prediction of cytotoxic T cell epitopes	http://crdd.osdd.net/raghava/ctlpred/	Default
13	IL-10Pred	Prediction of IL-10 inducer epitopes	http://webs.iiitd.edu.in/raghava/il10pred/predict3.php	Default
14	TAPPred	Prediction of TAP binding affinity of epitopes	http://crdd.osdd.net/raghava/tappred/	Default
15	IFNepitope	Prediction of IFN-γ inducer epitopes	http://crdd.osdd.net/raghava/ifnepitope/predict.php	Default
16	PEP-FOLD 3	Peptide and miniprotein structure prediction	http://bioserv.rpbs.univ-paris-diderot.fr/services/PEP-FOLD3/	Default
17	PatchDock	Molecular docking algorithm based on shape complementarity principles	https://bioinfo3d.cs.tau.ac.il/PatchDock/	Default
18	FireDock	Refinement and re-scoring of rigid-body protein-protein docking	http://bioinfo3d.cs.tau.ac.il/FireDock/	Default
19	ClusPro	Molecular docking	https://cluspro.bu.edu/login.php	Default

### Prediction of T cell epitopes

The subsequent step predicts the presence of immunogenic T cell epitopes in all the 5 GDPAA and 5 RSKMA by employing consensus technique of MHC class I and II tools accessible at IEDB web server. The prediction of T cell epitopes were performed for 51 HLA class I (cut off threshold IC_50_ ≤ 500 nM) and 54 HLA class II alleles (cut off threshold ≤ 3 percentile rank) [35]. These T cell epitopes were further screened by VaxiJen2.0 tool that predicted the antigenic epitopes with threshold ≥ 0.5 [32].

### Population coverage analysis of predicted T cell epitope ensemble

In the following step, PPC analysis of aforesaid combined (MHC class I and II) epitopes for all 5 GDPAA were conducted by utilizing IEDB based PPC tool against world-wide population. Furthermore, the minimal combined epitope set for each antigen was formed along with the inclusion of only those epitope which have highest PPC value and restricted by different set of MHC alleles. In case, PPC value of epitopes are equal then epitope with highest VaxiJen score was included in the minimal epitope set. Then, after employing the same protocol of minimal epitope selection, an ultimate epitope ensemble was designed with the joint screening of minimal epitope set of all the 5 GDPAA. Finally, the PPC of 5 GDPAA minimal epitope set and epitope ensemble were executed for the selected malaria endemic regions in the present study (India, South America, South-east Asia, Central Africa, East Africa, North Africa, South Africa and West Africa). So as to compare the predictive efficiency of PPC analysis for GDPAA epitope ensemble selection, the same protocol was also applied to predict epitope ensemble of 5 RSKMA and 151 EKE of *P. falciparum* [19, 36].

### Prediction of immunogenic induction

The immunogenicity of the MHC class I epitope ensemble designed from GDPAA was estimated by CTLPred and TAPPred [34, 37, 38] while induction of IFN-γ and IL-10 by MHC class II epitope ensemble from GDPAA were predicted using tools IFNepitope and IL-10Pred, respectively [39, 40].

### Prediction of epitope structure and docking studies

A structure-based docking approach was further carried out so as to improve the predictive capability of peptide-MHC binding. The combination of sequence and structure-based approaches not merely enhances the probability of MHC binding prediction but also calculates the docked epitope orientation. The complex crystal structures of HLA–A*02:01 (PDB ID: 1I4F) and HLA–DRB1*01:01 (PDB ID: 1AQD) were retrieved from the protein data bank (PDB) for MHC class I and class II, respectively. We used peptides NQMIFVSSI (C1) and LKELIKVGLPSFENL (C2) from EKE of *P. falciparum* as positive controls in docking studies. The structural information about the test peptides of GDPAA epitope ensemble (P1, P3 and P11) and positive control peptides were modelled using the PEP-FOLD 3 web server. The test peptides of GDPAA epitope ensemble were then docked by employing PatchDock web server to HLA–A*0201 and HLA–DRB1*0101 interacting residues as input. The best 10 HLA-peptide complexes were further refined by the FireDock web server. In order to compare the study of molecular docking, ClusPro tool was also used for the same test and control peptides along with target MHC molecule of interest [41]. The details of the docking web servers are given in the Table 1.

## Results and discussion

### Prediction and characterization of antigens

Primarily, 5548 proteins of *P. falciparum* genome were screened for the presence of signal peptide/ anchor using SignalP4.0 and found 640 surface accompanying/screatory proteins. Out of 640, only 17 are predicted to contain GPI-anchors using PredGPI tool and further 5 proteins are predicted as malarial adhesins and adhesin-like proteins. These 5 malarial adhesin proteins viz. CSP, surface protein P113 (P113), merozoite surface protein 1 (MSP1), 28 kDa ookinete surface protein (P28) and 25 kDa ookinete surface antigen precursor (P25), were also predicted as antigenic using VaxiJen, which contain ≤ 1 transmembrane helix as predicted by TMHMM2.0 server (Table 2). Among these malarial adhesin proteins, CSP is recognized as multifunctional protein that needed towards the sporozoite formation so as to mediate the parasite passage from vector mosquito midgut into the hepatocyte cells [42]. On the other hand, P113 is surface protein that expressed in almost every stage of parasite pertaining to its life-cycle. Such surface protein not only facilitates effective transformation of sporozoite in liver stage but also serve as a molecular link between the parasite and an erythrocyte [43, 44]. Likewise, MSP1 is an extremely abundant protein that covers the merozoite surface in almost all the species of malaria parasites and important with reference to invasion of erythrocyte [45]. In addition, P25 as well as P28 that behave as post-fertilization proteins are expressed in large quantities upon the surfaces of zygotes including the maturing ookinete stage of the parasite, which can likely to facilitate parasite clustering [46]. Interestingly, these GPI-anchored surface linked antigens are projected as promising vaccine targets against various developmental stage of the *P. falciparum* owing to their significant roles not only in the invasion of host cell but also in the completion of life-cycle of parasite as reviewed by Draper group [47]. These antigens are extremely conserved amongst *Plasmodium* species as well as in various isolates of different geographical region. Such important characteristics lead to the development of simpler malaria vaccine as single target gene sequence will be efficient against various parasite species like *P. falciparum, P. vivax* and *P. berghai.* Furthermore, a similar analysis data are also stored in MalVac database, which presently accessible as a scientific community resource for only 161 adhesin proteins belonging to *P. falciparum* [48].

**Table 2:** The predicted physicochemical properties of 5 genome derived predicted antigenic adhesins (GDPAA) and 5 randomly selected known malarial adhesins (RSKMA)

S.No.	PlasmoDB ID	Name of protein	No. of amino acids	GRAVY	pI (pH)	Water Solubility
GDPAA						
1	PF3D7_0304600	CSP	397	−1.249	5.36	Good
2	PF3D7_0930300	MSP1	1720	−0.655	6.11	Good
3	PF3D7_1030900	P28	218	−0.046	5.94	Poor
4	PF3D7_1031000	P25	217	−0.001	6.55	Good
5	PF3D7_1420700	P113	969	−1.063	4.48	Good
RSKMA						
1	PF3D7_0617400	Erythrocyte membrane protein 1, PfEMP1	2394	−1.013	6.10	Good
2	PF3D7_0616500	TRAP-like protein	1371	−1.183	5.12	Good
3	PF3D7_1133400	Apical membrane antigen 1	622	−0.812	5.36	Good
4	PF3D7_0731500	Erythrocyte binding antigen-175	1502	−1.093	5.52	Good
5	PF3D7_0202000	Knob-associated histidine-rich protein	654	−1.432	9.17	Good

**Fig. 2:**
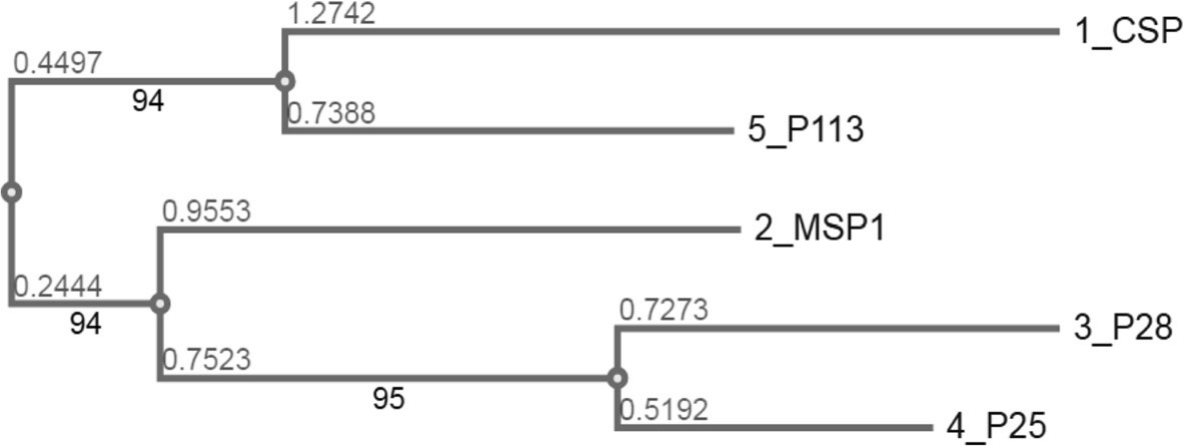
The clustering tree of 5 genome derived predicted antigenic adhesions (GDPAA) of *P. falciparum* generated by web server MAFFT version 7 with 100 times bootstrapping. The numbers above the branch showing branch length while below the branch showing bootstrap values

The appropriate physico-chemical properties as well as stable structure of proteins are needed to trigger immune response [33, 49]. The negative Gravy values of 5 GDPAA clearly indicate their hydrophilic nature and good water solubility except P28 (Table 2). The clustering analysis of these antigens also revealed a close similarity between CSP and P113 as well as P28 and P25 inferred by MAFFT7.0 web server (Fig. 2). A vaccine involving a little number of alleles may be enough for coverage towards naturally-circulating strains, which also supports the concept of multi-allele methodology for designing polymorphic antigens as malaria vaccines [50, 51]. The study conducted by Soulama group revealed that AMA-1 antigen of *P. falciparum* 3D7 showed lower allelic diversity in Central Africa compared to West Africa [52]. Thus, the limited allelic diversity of antigens screened in the present study is suitable vaccine candidates for further immunological experiment.

### Prediction of antigenic T cell epitopes

It would be a noticeable option to provoke sufficient T cell response that may enhance the protective efficacy as a result of additive/ synergistic effects [53, 54]. Considering this, the ultimate goal of the current investigation focused on integrating the epitopes belonging to aforementioned antigens presented by common human MHC class I and II molecules. Apart from this, the task of epitope discovery with vaccine designing is gradually dependent on bioinformatics tools and access to data relevant to immune reactions of specific pathogens. The tools involve validated and benchmarked methods to predict T cell epitopes of MHC class I and II binding peptides [36]. Furthermore, the protective efficacy of the vaccine is dependent on the polymorphic human MHC alleles and malaria antigens [55]. A total of 2647 T cell epitopes (755 HLA class I and 1892 HLA class II) were predicted for all the 5 GDPAA which were further screened by VaxiJen tool with threshold ≥ 0.5 resulted into a manageable 1270 antigenic epitopes (Table 3, Additional file 2) [32]. A similar data of T cell epitopes prediction for RSKMA are also presented in Table 3 and Additional file 3.

**Table 3:** Predicted number of T cell epitopes against 5 genome derived predicted antigenic adhesins (GDPAA) and 5 randomly selected known malarial adhesins (RSKMA)

S.No.	PlasmoDB ID	Total no. of predicted epitopes			No. of epitopes with VaxiJen score ≥ 0.5		
		MHC class I	MHC class II	Combined	MHC class I	MHC class II	Combined
GDPAA							
1	PF3D7_0304600	55	110	165	28	46	74
2	PF3D7_0930300	426	1005	1431	197	466	663
3	PF3D7_1030900	62	122	184	28	65	93
4	PF3D7_1031000	48	122	170	32	66	98
5	PF3D7_1420700	164	533	697	82	260	342
RSKMA							
1	PF3D7_0617400	485	1167	1652	238	427	665
2	PF3D7_0616500	299	796	1095	137	408	545
3	PF3D7_1133400	183	364	547	94	188	282
4	PF3D7_0731500	326	714	1040	175	427	602
5	PF3D7_0202000	110	167	277	58	99	157

### Population coverage analysis and selection of minimal epitope ensemble

**Fig. 3:**
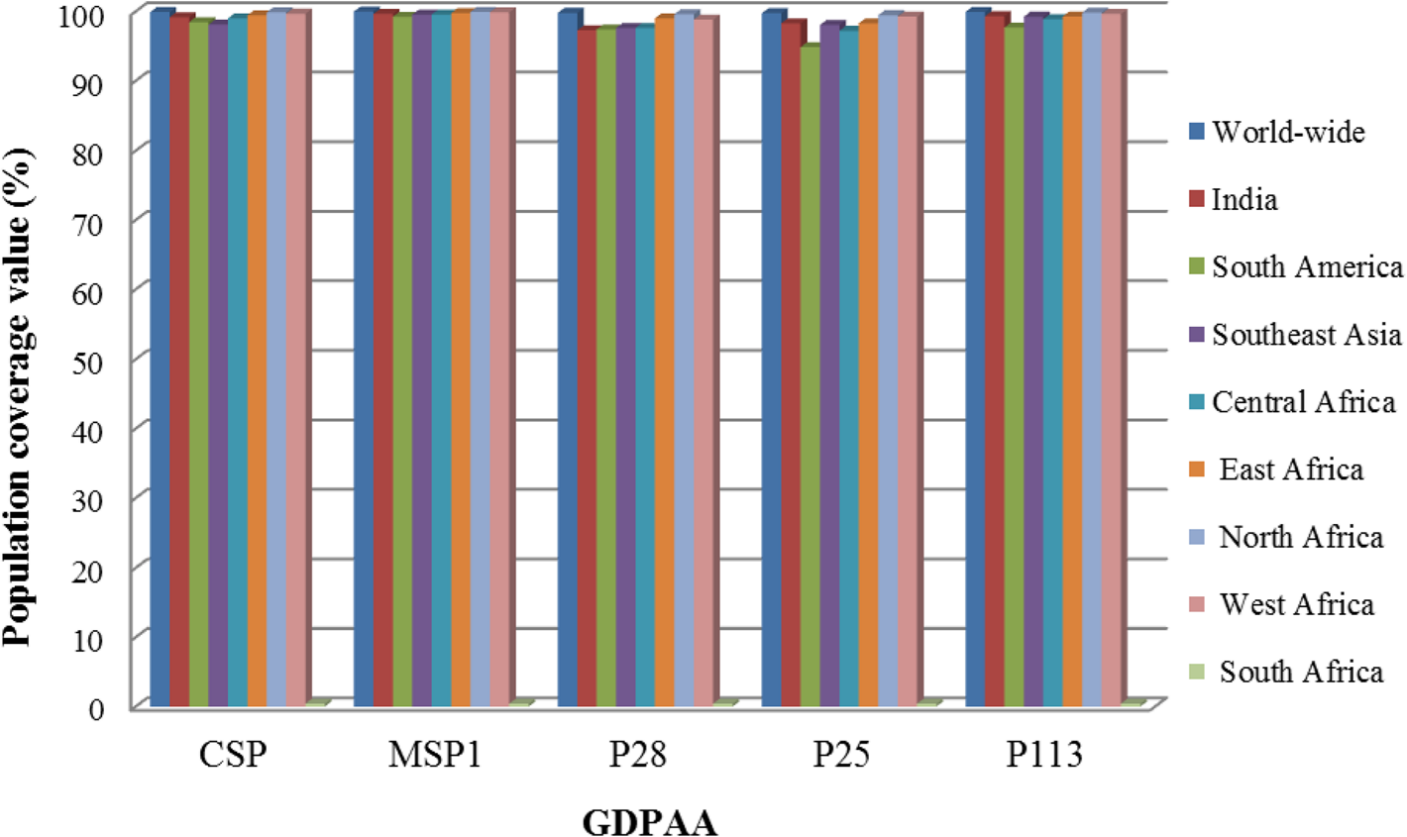
The predicted population coverage (PPC) of combined (MHC class I and II) T cell epitopes for 5 genome derived predicted antigenic adhesins (GDPAA) with VaxiJen score ≥ 0.5

The immunogens based on population coverage analysis enable us towards the screening of best possible minimal epitope combination, i.e., epitope ensemble so as to maximize vaccine effectiveness. In this background, the PPC strategy was followed for developing the epitope ensemble of chosen antigens towards improving the vaccine efficacy in the world-wide as well as endemic population of malaria. Primarily, the PPC of predicted T cell epitopes (MHC class I, II and combined) from 5 GDPAA with VaxiJen score ≥ 0.5 were performed for the world-wide, South East Asia, India, South-America and Central Africa, East Africa, North Africa and West Africa populations (Additional file 4). The minimum PPC value of combined epitopes (94.84%) for P25 is reported for South-America population, whereas the maximum PPC (99.99%) is forecasted for MSP1 antigen for world-wide population (Fig. 3) [36]. However, the PPC values of EKE of *P. falciparum* for 121 MHC class I, 30 MHC class II and 151 combined epitopes were found 96.97, 65.35 and 99.25%, correspondingly. In order to minimize the epitopes in the vaccine construct design, we have restricted epitopes number on the basis of considering minimal epitopes binding with maximum number of different MHC alleles for the entire 5 GDPAA (Additional file 5). This resulted into 5 minimal set of epitopes covering populations world-wide, South East Asia, India, South America, Central Africa, East Africa, North Africa and West Africa (Additional file 6). The PPC value (86.18%) of combined minimal epitopes is minimum for CSP antigen for South-East Asian population while the maximum coverage (99.95%) for MSP1 antigen is reported for world-wide population. Further, employing the above protocol of minimal epitope selection, an epitope ensemble was designed for each of the combined minimal epitopes of 5 GDPAA and 5 RSKMA as well as 151 EKE. Here, as detailed in Table 4, the PPC value of GDPAA epitope ensemble (99.97%) (Additional file 6) is significantly higher over the epitope ensemble of RSKMA (99.90%) (Additional file 3) and EKE of *P. falciparum* epitope ensemble (97.72%) for the world-wide population (Table 4). Also, it is interesting to note that the PPC value of predicted MHC class II epitope ensemble of GDPAA (93.71%) is much higher than the MHC class II epitope ensemble of EKE of *P. falciparum* (75.31%). However, an exception was recorded for South African population (0.43%) that could be due to limited HLA typing data as detailed in Table 5.

**Table 4:** The world-wide PPC coverage of epitope ensemble predicted from all 5 genome derived predicted antigenic adhesins (GDPAA) and 5 randomly selected known malarial adhesins (RSKMA) as well as 151 experimentally known epitopes (EKE) of *P. falciparum*

S.No.	Epitope ensemble	PPC for world-wide population (%)		
		MHC class I	MHC class II	Combined
1.	GDPAA MHC class I: FAMSNALLV, ILMLILYSF, YTLTAGVCV, FSSSNNSVY, YEMKFNNNF, LIVCSIFIK, YFNDDIKQF, LLKSYKYIK, SRLKKRKYF, KGMSSSQEM MHC class II: IPFFILHILLLQFLL, FIQLYITLNKARVTE, TCGNGIQVRIKPGSA	99.53	93.71	99.97
2.	RSKMA MHC class I: YFFASFFVL, FTYDSEEYY, FAFPPTEPL, FMPPRRQHF, FRDEWWKVI, RIYDKNLLM, KLYFPTPAL, YAFSEECPY, ISFQNYTYL MHC class II: DKMKIIIASSAAVAV, YKYAASFTLAAILFL	98.67	92.31	99.90
3.	EKE of *P. falciparum* MHC class I: RPRGDNFAV, TPYAGEPAPF, YLINKHWQR, NQMIFVSSI, KVSDEIWNY MHC class II: DAEVAGTQYRLPSGKCPVFG, LKELIKVGLPSFENL, ALLIIPPKIHISIEL	90.78	75.31	97.72

**Table 5:** The PPC value of epitope ensemble vaccine candidate of 5 genome derived predicted antigenic adhesins (GDPAA) and 5 randomly selected known malarial adhesins (RSKMA) as well as 151 experimentally known epitopes (EKE) of *P. falciparum* for malaria endemic regions

Population	GDPAA (%)	RSKMA (%)	EKE of *P. falciparum* (%)
North Africa	99.93	99.84	95.34
India	99.54	99.17	93.01
East Africa	99.68	99.41	92.17
Southeast Asia	99.39	99.07	81.71
South America	98.94	97.96	87.66
Central Africa	99.31	98.78	90.47
West Africa	99.84	99.19	92.88
South Africa	0.43	0.43	0.40

The GDPAA epitope ensemble as a vaccine candidate includes 10 MHC class I epitopes (P1:FAMSNALLV, P2:ILMLILYSF, P3:YTLTAGVCV, P4:FSSSNNSVY, P5:YEMKFNNNF, P6:LIVCSIFIK, P7:YFNDDIKQF, P8:LLKSYKYIK, P9:SRLKKRKYF, P10:KGMSSSQEM) and 3 MHC class II epitopes (P11:IPFFILHILLLQFLL, P12:FIQLYITLNKARVTE, P13:TCGNGIQVRIKPGSA) as presented in Table 4 (Additional file 7). Out of 10 MHC class I epitopes, 4 epitopes (P1, P2, P5 and P9) were confirmed as cytotoxic T-lymphocyte (CTL) epitopes by using CTLPred, while 7 epitopes (P1, P2, P3, P5, P7, P8, and P9) were confirmed as TAP binders by TAPPred. Also, the epitope P11 was found to be inducer of both IFN-γ and IL-10 cytokines predicted by IFN-epitope and IL-10Pred, respectively while epitope P13 induced only IFN-γ. The GDPAA epitope ensemble showed 99.54% of PPC value for Indian population (Table 5). In the West African population, HLA–B*5301 and HLA–DRB1*1302 alleles are linked with the reduction in life-threatening malaria [56]. In our study, the GDPAA epitope ensemble P1: FAMSNALLV binds to the HLA–B*5301 and P12: FIQLYITLNKARVTE also binds to the HLA–DRB1*1302. Moreover, the epitope P4: FSSSNNSVY and P8: LLKSYKYIK of GDPAA ensemble binds to HLA–A*30:02 and HLA–A*30:01, correspondingly, which are also linked with cerebral malaria [57]. In addition, the MHC class II epitopes P12: FIQLYITLNKARVTE and P11: IPFFILHILLLQFLL binds to the HLA–DRB1*04:01, which is accompanying with severe malaria in Northern Ghana [58]. There are 5 most frequently HLA alleles (HLA–B*15:03, HLA–B*42:01, HLA–B*53:01, HLA–B*58:02 and HLA–B*57:03) found in African regions [59]. The peptide P10: KGMSSSQEM found in the GDPAA epitope ensemble also binds to HLA–B*5801, while peptide P1: FAMSNALLV binds to HLA–B*5301.

**Fig. 4:**
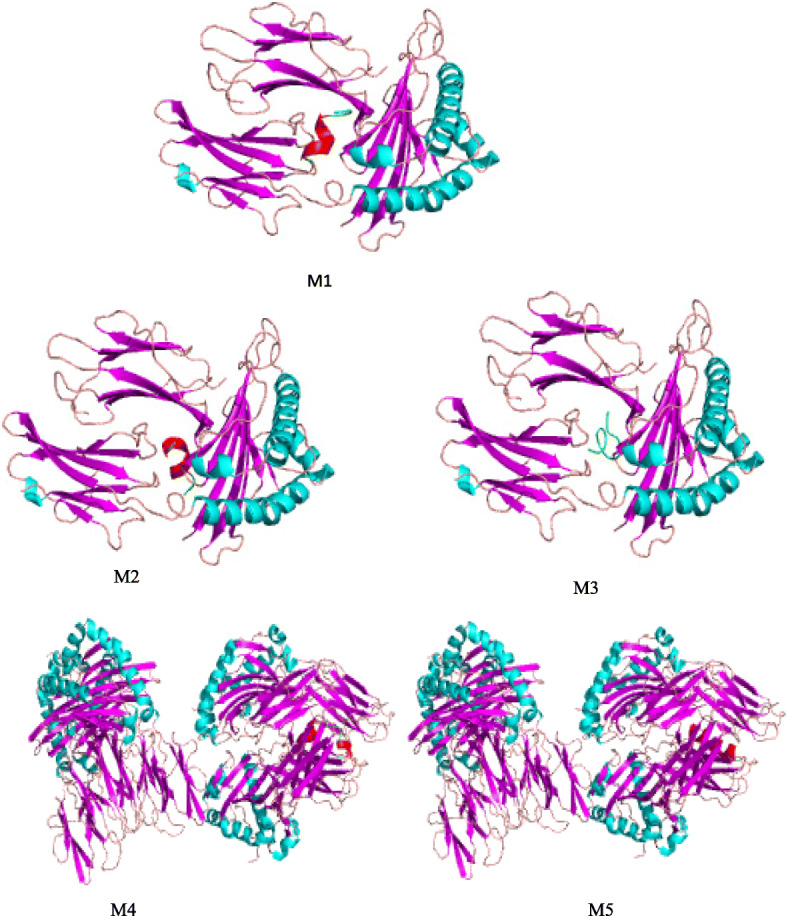
The visual models (M1-M5) of the best docking orientation as predicted by ClusPro for the HLA–A*0201 and HLA–DRB1*0101 molecules complexed with test epitopes (P1 and P3) and control epitopes (C1 and C2). M1: complex of epitope C1 with HLA–A*0201, M2: complex of epitope P1 with HLA–A*0201, M3: complex of epitope P3 with HLA–A*0201 M4: complex of epitope C2 with HLA–DRB1*0101 and (e) M5: complex of epitope P11 with HLA–DRB1*0101. The epitope in models are shown in red and green colour

The interaction between the peptide and MHC is an important characteristic of T cell epitope and the accessibility of the crystal structures facilitates us to determine the mode of interaction by computational methodology [60]. The docking data obtained from the freely available online tools PatchDock and ClusPro depicted that the anchor residues of majority of the epitope ensemble associate with the active site residues of HLA–A*0201 and HLA–DRB1*0101 that specify the superior mode of binding of screened epitopes with the T cell receptor. For HLA–A*0201, the geometric shape complementarities scores for test peptides P1 and P3 were 7284 and 7586, respectively, which were very close to the score of the positive control peptide C1 (7604) and global energy for test peptides P1and P3 were − 11.93 and − 15.72, correspondingly that were less than to the positive control peptide C1 (− 7.99). Similarly for HLA–DRB1*0101, the geometric shape complementarity scores for P11 was 11,870, which was very close to the score of the positive control C2 peptide (10806) and global energy for P11 was − 61.82, which was less than the positive control C2 peptide (− 55.11). The ClusPro docking tool was also used to predict the HLA–epitope complex molecular model and calculate the clustering score. The test epitopes (P1, P3 and P11), target MHC proteins and control peptides were same as selected in the PatchDock tool to compare the docking results. For HLA–A*0201, the lowest clustering scores for P1 and P3 peptides were − 694.4 and − 758.3, respectively, which were lower to the score of the positive control C1 peptide (− 647.1). Similarly, for HLA-DRB1*0101, the lowest clustering score for P11 peptides was − 1159.8 which was very much lower than the score of the positive control C2 peptide (− 715.7). The docking models for HLA–A*0201-C1, HLA–A*0201-P1, HLA–A*0201-P3, HLA–DRB1*0101-C2 and HLA–DRB1*0101-P11 are shown in the Fig. 4.

The reverse vaccinology and immunoinformatics strategies are still under progressive phase, however reasonable triumphs may result in substantial advancement on epitope-based ensemble vaccine efficacy against malaria pathogens for example, by enhancing coverage in the target populations through reasonably bearing in mind the specificity as well as occurrence of the HLA molecules [61, 62]. Therefore, epitope ensemble provided in the present study provides the basis for effective malaria vaccine design. A well-known drawback of epitope ensemble vaccine is poor immunogenicity, usually necessitating the use of suitable adjuvants [63]. Therefore, these predicted and experimentally validated epitopes ensemble could be tested for further studies like nanovaccine formulation and evaluation in the experimental animal model for actual efficacy of nano sized malaria vaccine. Numerous investigations found to depict that malarial antigens showed more immunogenicity and superior correlated with protection when presented on nanoparticles based carrier systems like self-assembling protein nanoparticle (SAPN). The SAPN depend on coiled-coil domains of proteins to form stable nanoparticles [64]. Recently, Burkhard and Lanar developed protein based nano vaccines, which provide robust immunity against malaria [65]. This SAPN contains HLA-supertypes-restricted CD8^+^ T cell epitopes (separated by N/KAAA spacers and optimized for proteasomal cleavage) from antigens expressed during malaria pathogen life-cycle, the universal CD4^+^ T cell epitope, and flagellin as a scaffold and TLR5 agonist. On the other hand, used de novo designed amino acid domains to fuel the development of the coiled-coil scaffolds that present the antigenic epitopes on the nanoparticles surface [66]. As the surface area of the nanoparticles increases with the reduction of particles size, therefore there is a great need to develop more in silico strategy for effective nanovaccine designs that fulfil the vaccine requirement of needy human being [67].

## Conclusions

The design of epitope ensemble using computational vaccinology is one of the promising alternatives, which enables finding of new epitope based vaccine candidate in a cost-effective manner for global as well as *P. falciparum* malaria endemic population. The present study screened 5 GDPAA as potential vaccine targets due to their extreme conservancy amongst *Plasmodium* species including isolates of different geographical region. The PPC analysis with respect to epitope ensemble of 5 GDPAA and 5 RSKMA as well as 151 EKE of *P. falciparum* showed more than 81% population coverage in the world-wide along with malarial endemic regions except South Africa. These promiscuous T cell epitope ensembles will significantly aid towards the fast development of more efficacious malaria vaccine against *P. falciparum.* Therefore, this promising strategy could be extended to other infectious diseases as well. Overall, the computational tools used here are not yet ready to substitute the wet laboratory experimentation, rather they are assisting in experimental design and reducing the time and cost of the vaccine development process.

## Additional files

Additional file 1: Experimentally known epitopes of *P. falciparum* 3D7. (XLSX 11 kb)

Additional file 2: **(a)** The PPC of predicted MHC class I binding T cell epitopes from 5 genome derived predicted antigenic adhesions (GDPAA) with VaxiJen score ≥ 0.5, **(b)** The PPC of predicted MHC class II binding T cell epitopes from 5 genome derived predicted antigenic adhesions (GDPAA) with VaxiJen score ≥ 0.5. **(**XLSX 88 kb**)**

Additional file 3: **(a)** The PPC of predicted T cell epitopes (MHC class I, MHC class II and combined) from 5 randomly selected known malarial adhesions (RSKMA) with VaxiJen score ≥ 0.5, **(b)** The PPC of predicted T cell epitopes (MHC class I, MHC class II and combined) from 5 RSKMA, **(c)** Predicted set of T cell epitopes of 5 RSKMA, **(d)** The PPC of minimal T cell epitopes (MHC class I, MHC class II and combined) from 5 RKSMA, **(e)** The predicted ensemble epitope (MHC class I and MHC class II) from 5 RKSMA. (XLSX 145 kb)

Additional file 4: The PPC of predicted T cell epitopes for 5 genome derived predicted antigenic adhesions (GDPAA). (XLSX 9 kb)

Additional file 5: The PPC of individual epitope found in minimal epitope set of 5 genome derived predicted antigenic adhesions (GDPAA). (XLSX 13 kb)

Additional file 6: The PPC of minimal combined epitope set for 5 GDPAA and jointly screened epitope ensemble. (XLSX 10 kb)

Additional file 7: The PPC value of individual epitope of epitope ensemble designed from GDPAA and experimentally known epitope (EKE) of *P. falciparum*. (XLSX 11 kb)

## Abbreviations

AMA-1: Apical membrane antigen-1
CSP: Circumsporozoite protein
CTL: Cytotoxic T-lymphocyte
GPI-anchors: Glycophosphatidylinositol-anchors
GRAVY: Grand average of hydropathicity
HLA: Human leukocyte antigen
IEDB: Immune epitope database
IFN: Interferon
IL-10: Interleukin-10
MHC: Major histocompatibilty complex
MSP: Merozoite surface protein
PDB: Protein data bank
PPC: Predicted population coverage
SAPN: Self-assembling protein nanoparticle
TAP: Transporter associated with antigen processing
WHO: World Health Organisation
